# Criteria of teacher and students's didactic performances in psychology: the Peruvian university students' perceptions

**DOI:** 10.3389/fpsyg.2026.1751054

**Published:** 2026-02-16

**Authors:** Aldo Bazán-Ramírez, Walter Capa-Luque, Carmela R. Henostroza-Mota, Luz E. Mayorga-Falcón, William Montgomery-Urday, Edwin D. Félix-Benites, Narciso A. Palomino-Miranda, Juan Solano-Gutierrez, Max H. Chauca-Calvo

**Affiliations:** 1 Department of Education and Humanities, Universidad Nacional José María Arguedas, Andahuaylas, Peru; 2 Faculty of Psychology, Universidad Nacional Federico Villarreal, Lima, Peru; 3 Faculty of Psychology, Universidad Nacional Mayor de San Marcos, Lima, Peru; 4 Faculty of Educational Sciences, Universidad Nacional del Callao, Callao, Peru; 5 Psychology School, Universidad Científica del Sur, Lima, Peru

**Keywords:** didactic performance, formative assessment, improvement-application, learning, teaching

## Abstract

**Introduction:**

Teacher-student interaction in teaching and learning situations of scientific discipline in the context of higher education presupposes the performances of both teachers and students. These performances are functionally related and are focused on achieving educational objectives.

**Method:**

This is a cross-sectional and predictive study that analyzes the effects of teacher's didactic performance criteria perceived by students on the self-assessment of student didactic performance criteria, examined through multiple linear regression models and path analysis with second-order variables and a mediator within the regression model. A total of 541 psychology students from a Peruvian public university (141 male and 400 female) participated, selected through non-probabilistic sampling. The student assessment scale on teacher didactic performance and the student didactic performance self-assessment scale were administered.

**Results:**

Significant covariation was found between each pair of teacher-student performance criteria. The first linear regression model (*R*^2^ = 0.54, *p* < 0.001) predicts student learning based on teaching and formative assessment; likewise, the second regression model (*R*^2^ = 0.61, *p* < 0.001) indicates that the formative assessment and teaching factors predicts students' capacity for improvement-application. In structural equation modeling with path analysis, the teacher factors Teaching and Formative Assessment have a direct effect on the Learning; and an indirect effect, mediated by Learning, on the factor Improvement-Application. In structural equation modeling with path analysis, the teacher factors “Teaching” and “Formative Assessment” have a direct effect on the Learning factor and an indirect effect on the Improvement-Application factor.

**Conclusion:**

The professor's teaching strategy had a direct impact on student learning. Likewise, the formative evaluation had a greater indirect effect on the improvement-application.

## Introduction

Teaching practice in the context of higher education involves a set of performances during didactic interactions with students. This teacher-student interaction in the teaching and learning situations of a discipline is configured through the performance of teachers and the performance of students, oriented toward the achievement of educational and instructional objectives. The identification and analysis of these functionally related performances have been the subject of research from various theoretical and methodological approaches.

Despite the nature of the functional teacher-student relationship during didactic interactions, interest has largely focused on the didactic performance of teachers in the context of higher education. Some researchers have focused on teaching strategies, for example, project methods, experimental methods, demonstration methods (Amber et al., 2023); student-centered teaching and learning approaches (Memon et al., 2023). Also, the importance of structural and practical improvements at the curricular level of university courses have been the subject of study (Kärkkäinen et al., 2023). Other studies have focused on the importance of quality feedback and building trusting relationships between teachers and their students (Shield, 2023).

On the other hand, Liang et al. (2023) identified two identity traditions of university teacher educators: a “master of masters” identity prevalent in provincial universities; and another “researcher” identity, prevalent in top-tier universities. Likewise, studies of observation of didactic performance in teaching-learning situations, using checklists and observational recording systems, have been reported at the middle and higher levels (Neves-Balan et al., 2022; Velarde-Corrales and Bazán-Ramírez, 2019).

An almost shared mode and common practice to identify the performance of university teachers is the use of self-reports (questionnaires or scales). As a widespread practice, students evaluate various features of the performance of their teachers who teach them (Alquraan et al., 2023; Bazán-Ramírez et al., 2023; Casillas et al., 2016; Chan, 2018; Eouanzoui and Jones, 2017; Feisthauer and Richter, 2018; Neves-Balan et al., 2022; Scherer and Gustafsson, 2015). Mathematical modeling and multivariate statistical analysis models have also been raised in the evaluation of university teachers' teaching practices (Li and Guo, 2024; Lu and Yi, 2024).

However, it has been reported that the measurement of teacher's didactic performance with student self-report scales is susceptible to variation according to discipline, student characteristics, and the type of study programs the students come from (Alquraan et al., 2024; Henriquez et al., 2023). These biases should be controlled with an analysis of invariance of student self-reported measures of teacher performance (Alquraan et al., 2023, 2024; Bazán-Ramírez et al., 2023; Henriquez et al., 2023). Along these lines, Cook et al. (2024) have proposed a model to control biases in the statistical analyses and in the cognitive components of students‘ evaluation of their teachers' performance. As already pointed out by Jornet et al. (2012), the validation of assessment instruments of non-cognitive variables that are associated with educational processes and outcomes must meet quality standards in the measurement of psychological and educational constructs.

Regarding Peru, (León 1978) investigated the inconveniences of the evaluation of teachers‘ performance by the student body and raised the issue of reciprocity in the students' evaluation of their teachers‘ performance, depending on the academic grade that their teachers give them in a course. (Arce-Saavedra and Blumen 2022) reported that the pedagogical practice of the teacher is significant because the performance is innovative, creative and because of the teacher's self-efficacy. Montes de Oca et al. (2023) found that the performance of psychology faculty at two universities in Lima, as perceived by students, and the learning strategies of the student body, have a significant impact on students' academic satisfaction. Bazán-Ramírez et al. (2022a) found that the didactic performance of a latent factor called “teacher teaching” had a significant direct effect on student performance in participation, while teacher performance in formative assessment had a significant impact on student performance in relevant practice and feedback.

Martínez et al. (2022) based on in-depth interviews with seven Peruvian university teachers, both in engineering and educational sciences, using the Atlas ti program and content analysis, derived three categories from the teachers' own assessment of teaching performance in formative assessment: the role of the teacher in formative assessment, the socio-emotional link and learning feedback. On the other hand, Ochoa et al. (2022) specified six dimensions of teaching performance: three in Knowledge Management competency (Appropriate planning, Generation of objectives and curricular content, and Appropriate methodology) and three in Technological competency (Management of information and communication technologies, Pedagogical resources, and Evaluation of learning). Taking a sample of graduate students from a private university in Lima, Ica and Huancayo, they reported that 72% of students rate the quality of their teachers as good, including knowledge management and technological competence.

Similarly, Cuellar-Quispe et al. (2023), in a study of perceptions of teaching performance conducted with 2,263 students from public and private universities in Peru, observed that teachers performed highly in five criteria of teaching performance: clarification of criteria, illustration, supervision of learning practices and activities, feedback, and assessment. They also observed that teachers showed high empathy with their students and good digital skills.

Bringas et al. (2023) identified that more than 50% of teachers rate themselves at an achieved level in the five dimensions evaluated: information, communication, content creation, safety and problem solving. In another study, Martín-Párraga et al. (2023) compared the performance of 808 university teachers in Seville (Spain) and 1658 university teachers in Arequipa (Peru), in dimensions such as professional commitment, digital resources, teaching and learning, assessment, student empowerment, and facilitation of student digital competence. Peruvian teachers rated their performance better than Spanish teachers. In Peruvian teachers, the teaching-learning and student empowerment competencies were the highest; whereas, in Spanish teachers, the digital resources and professional commitment competencies were the highest. Rojas-Osorio et al. (2024) evaluated the self-report of 112 Peruvian teachers from a private university on their digital teaching competencies, using 16 performance dimensions. They concluded that teachers‘ gender and age significantly influence teachers' digital competencies.

Bazán-Ramírez et al. (2025) compared the self-rating of 64 university teachers from a public university in northwestern Mexico and 139 university teachers from two public universities in Peru, on seven criteria (dimensions) of didactic performance, in the careers of Psychology and Education, with respect to two didactic moments or episodes. Teaching, with four didactic performance criteria: didactic planning, competence exploration, explicitness of criteria and illustration. Formative assessment, with three didactic performance criteria: supervision of practices, feedback and evaluation. At the level of didactic performance criteria, the performances of instructional planning and explicitness of achievement criteria were the most valued by the teacher. With respect to performance by didactic episodes or moments, significant differences were found in both teaching and formative evaluation, by discipline of origin (in favor of educational sciences) and gender of the teacher, in favor of women.

Capa-Luque et al. (2025), when examining the effects of teacher's didactic performance criteria on the didactic performance criteria of psychology students, found that the performance criteria used by teachers have varying degrees of direct and indirect impact on the student performance criterion Evaluation-application. Through a multiple causal chain mediation analysis, the authors also found that application-transfer competence in psychology students follows two indirect routes. The first route shows that Formative Assessment plays a mediating role in the causal relationship between Teaching and Improvement-Application; the second route involves the presence of double mediation (Formative Assessment and Learning) in the indicated causal relationship.

### Interbehavioral model of didactic performance

In this research we have assumed as substantive theory, the interbehavioral model of didactic performance (Carpio et al., 1998; Irigoyen et al., 2011a,b; Silva et al., 2014; Velarde-Corrales and Bazán-Ramírez, 2019), which was developed based on the psychological field model in educational contexts from the proposals and development of authors such as (Kantor 1959, 1975); Kantor and Smith (1975); (Ribes 1993, 2008) and Ribes et al. (1996).

Based on theoretical approaches and empirical studies derived from the perspective of interbehavioral psychology, seven criteria or areas of teaching performance have been proposed for teachers: lesson planning, competency exploration, clarification of criteria, illustration, supervision of practices, feedback, and evaluation (Bazán-Ramírez et al., 2025; Silva et al., 2014). Through questionnaires assessing students' opinions of their teachers' teaching performance (Bazán-Ramírez et al., 2023) and teachers' self-assessments of their own teaching performance (Bazán-Ramírez et al., 2025). Using structural modeling with latent variables from second-order confirmatory factor analysis, two constructs of interactive episodes (didactic interactions) were generated and validated. One focused on teaching-related performances and the other on formative assessment performances. These latent variables are in concordance with John Carroll's (1963) psycho-pedagogical model of teaching and learning. These instructional processes, teaching quality, curricular coverage, cognitive and motivational variables of the student body, and formative assessment influence learning achievement and academic outcome (Elliott, 2015; Hwang and Ham, 2021; Schmidt and Burroughs, 2013).

Considering these didactic performance criteria of the teacher and student, it is pertinent to deepen in studies that allow describing and explaining the relationship between each pair of didactic performance criteria teacher-student, according to the student's assessment, using self-report instruments with satisfactory construct validity.

### Questions, objectives and hypotheses

The first research question that has been posed is the following: ¿How do the criteria for didactic performance as perceived by students relate to the criteria for student performance in psychology classes at a public university in Peru?

**Figure 1:**
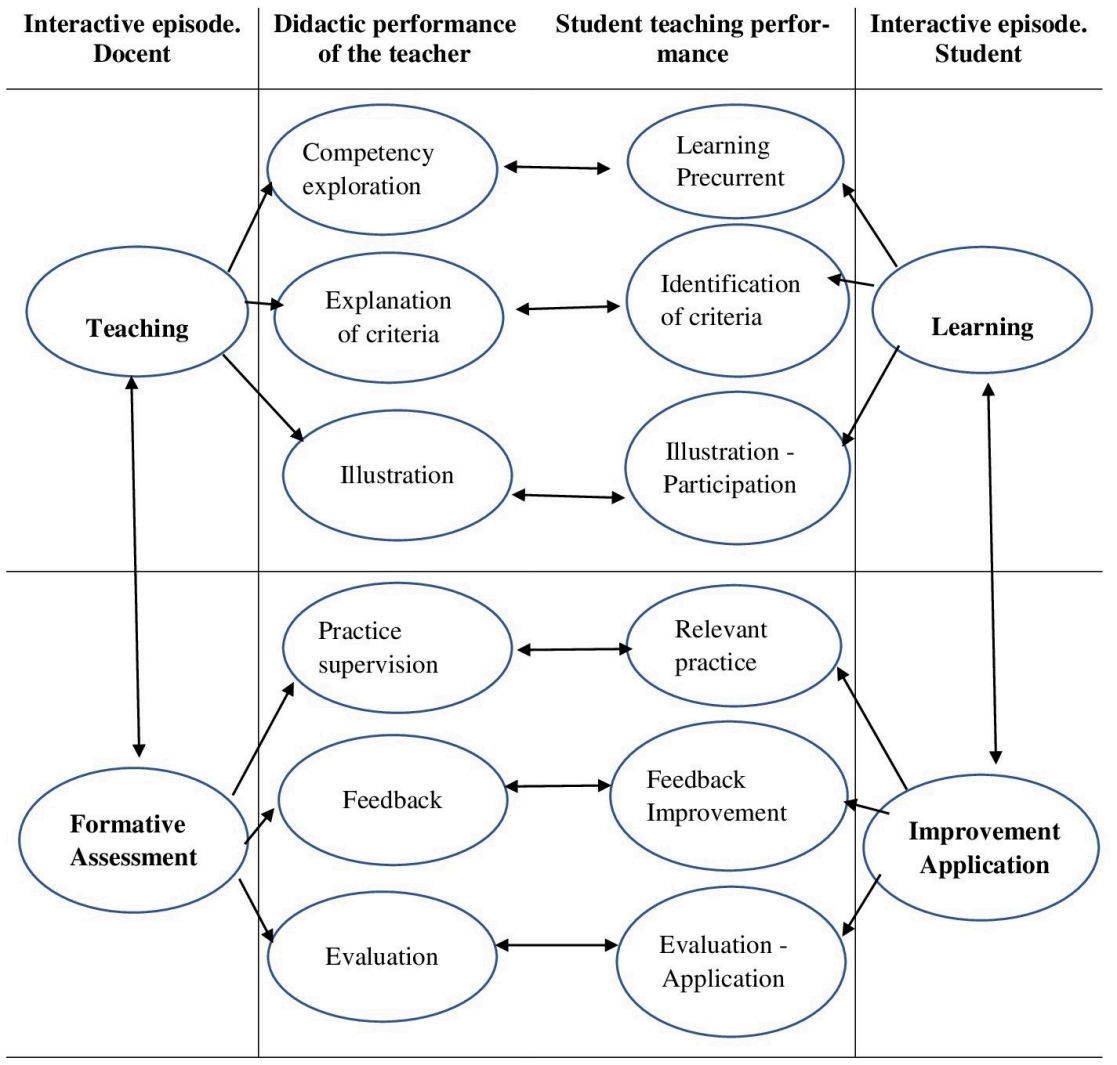
Relationship between teacher and student didactic performance, according to student self-report.

To answer this question, Figure 1 illustrates the hypothetical model to be tested. The didactic interaction is divided into two episodes or moments: one of teaching and the other of formative evaluation (and their pairs: learning and improvement-application). However, it should be understood that all criteria are functionally interrelated (Bazán-Ramírez et al., 2025). Consequently, the objective of this study was to determine, through latent correlations, the correspondence between students' perceptions of teachers' didactic performance and students' self-assessment of their own didactic performance. To achieve this objective, the construct validity and reliability of two self-reports (the scale for students to assess the didactic performance of the teacher and the scale for students to self-assess their own didactic performance) were first obtained.

**Figure 2:**
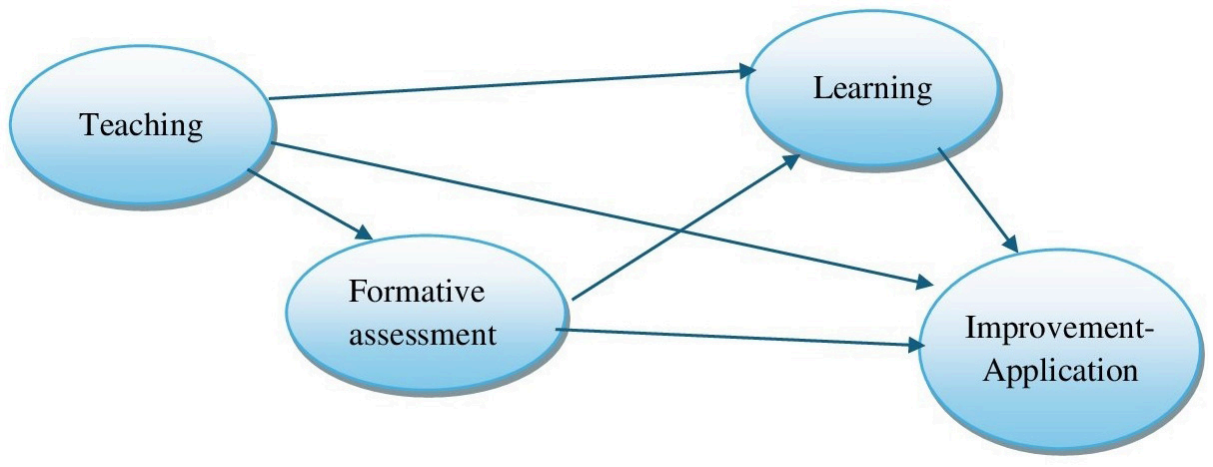
Structural regression model of teacher-student didactic performance.

The second research question was posed as follows: How do the effects of the constructs Teaching and Formative Assessment, as perceived by students, on the constructs Learning and Improvement-Application, as self-assessed by psychology students? The objective was to determine the effects of second-order variables of teacher didactic performance on second-order variables of student didactic performance in psychology classes at a public university in Peru. To this end, Figure 2 presents the hypothetical structural regression model of teacher-student didactic performance.

## Method

### Participants

A basic research study was conducted with a non-experimental design, cross-sectional measurement using two self-report scales. Data was collected from 541 students who took classes between May and August 2023. Table 1 describes the sample taken. The complete groups were taken from the classrooms whose teachers had agreed to participate in this study. Out of a total of 83 teachers in the 2023-II cycle, 51 teachers accepted. The sampling used was non-probabilistic convenience sampling, for both students and teachers of the Faculty of Psychology of a public university located in Lima (Peru).

**Table 1:** Sample distribution.

	**Variable**	**Categories**	***Fr***	***%***
Students	Gender	Male	141	26.06%
(*n* = 541)		Female	400	73.94%
	Cycle	I (1^st^ cycle)	187	34.57%
		III (3^rd^ cycle)	174	32.16%
		V (5^th^ cycle)	127	23.48%
		IX (9^th^ cycle)	21	3.88%
		XI (11^th^ cycle)	32	5.92%

Fr: frequency, %: relative share.

### Instruments

Two student rating scales on didactic performance were used, validated with Peruvian students of biological sciences (Bazán-Ramírez et al., 2023) and with Peruvian graduate students in educational sciences (Bazán-Ramírez et al., 2022b). The first scale evaluates the students' perception of their teachers' didactic performance. It is made up of 24 items, four for each of six dimensions of teacher didactic performance: competency exploration, identification of criteria, illustration, supervision of practice, feedback and evaluation. The second scale evaluates the students' self-assessment of their didactic performance. It consists of 24 items, organized into six subscales or dimensions: pre-current for learning, identification of criteria, illustration-participation, relevant practice, feedback-improvement and evaluation-application. Each criterion or dimension presents four items with graded responses (never = 0, Almost never = 1, almost always = 2, always = 3). Appendix 1 presents both scales for the evaluation of the didactic performance.

### Ethical considerations

The initial project was evaluated by the research and ethics committee of the UNFV. It was approved by Resolution N° 1529-2023- CU- UNFV. Subsequently, after authorization from the Director of the Department of Psychology of the University where the study was conducted, all teachers were invited to participate in the project by e-mail and in person. They were informed about the procedure, results and benefits of this research for the academic community of the university and for the improvement of the training process of psychologists. Each teacher who agreed to participate signed the informed consent form. Once the teachers‘ consent was obtained, each teacher's classroom was entered to inform the students about the research process and the benefits of the study, as well as the students' right to participate in the study or leave it when they considered it pertinent. Each student also signed the informed consent form.

### Data analysis procedure

Based on the construct validity results of both scales, two types of analysis were performed, according to the questions posed. In the first case, using the RStudio program and Lavaan package, latent correlations were performed with structural equation modeling to test six hypothetical models, considering each pair of the six teacher-student teaching performance criteria as latent variables.

**Figure 3:**
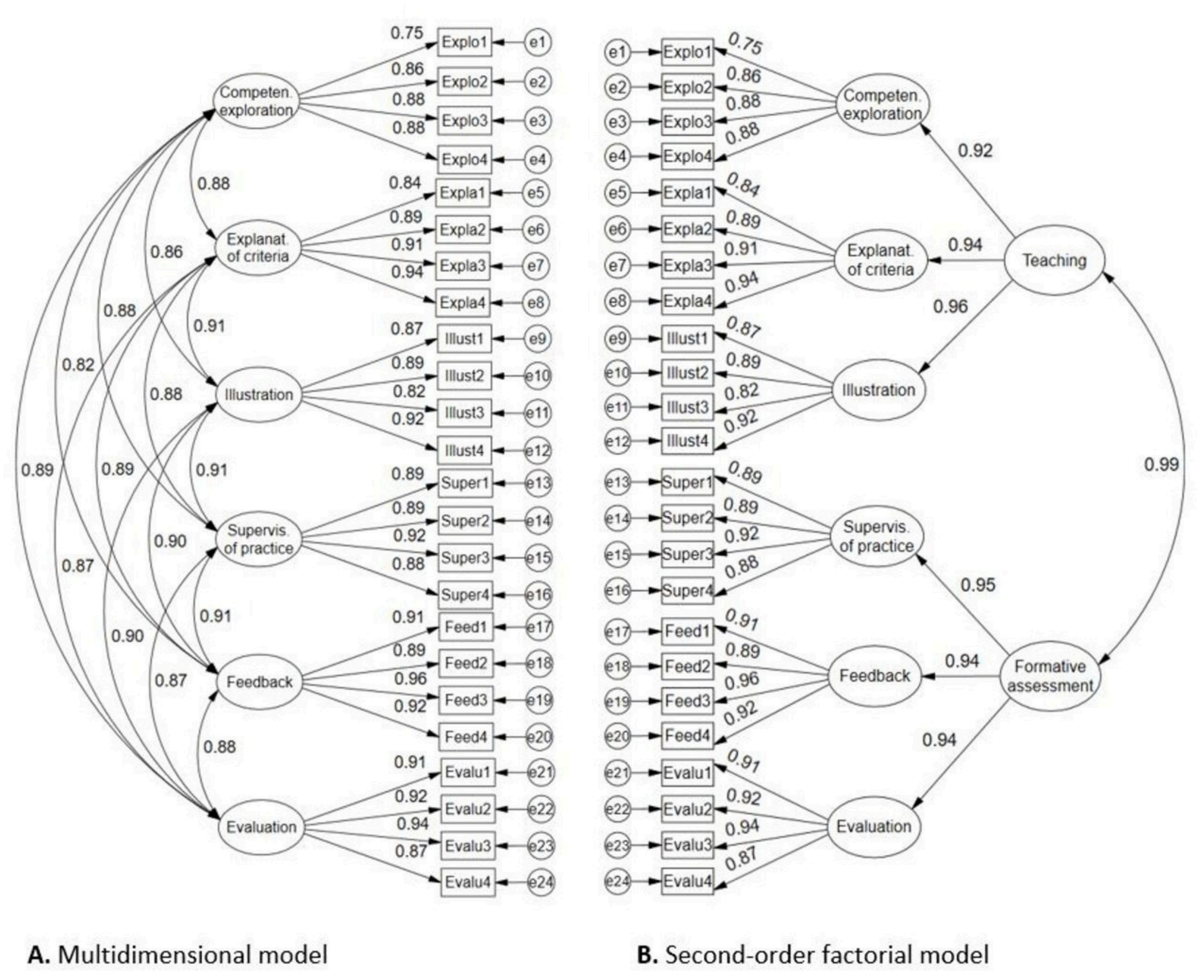
Factor structures of student assessment scale on the teacher's didactic performance. **(A)** Multidimensional model; **(B)** Second-order factorial model.

**Figure 4:**
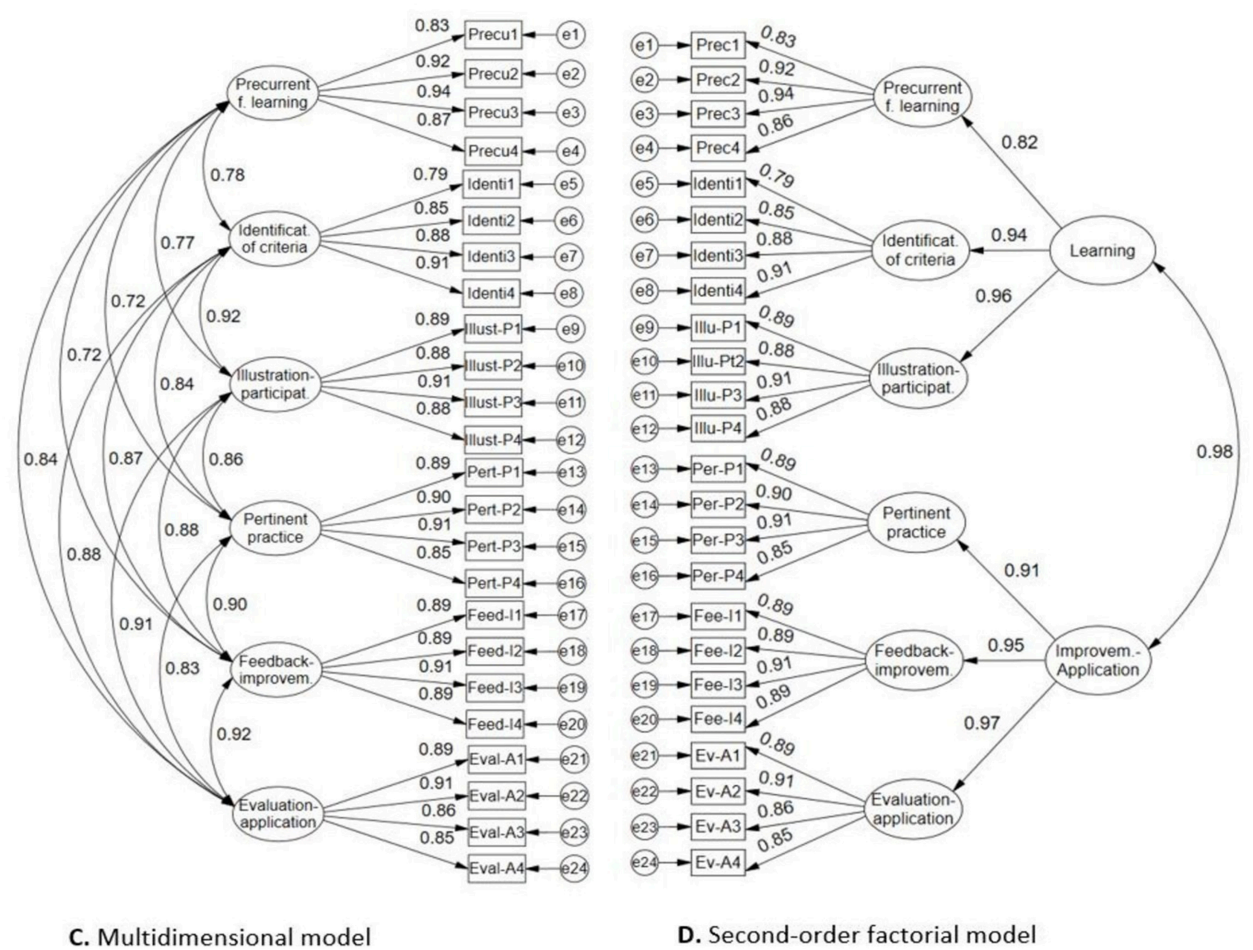
Factor structures of student assessment scale on the teacher's didactic performance. **(C)** Multidimensional model; **(D)** Second-order factorial model.

In the second case, taking into account the resulting second-order CFA models shown in Figures 3, 4, using RStudio software, a path analysis hypothetical model was tested with structural equation modeling for the second-order factors transformed into manifest variables (teaching, formative assessment, learning, and improvement-application), taking into account the scheme in Figure 2 described above.

## Results

### Validity and reliability of the student rating scale on teacher didactic performance

Figure 3 shows the results of the confirmatory factor analysis for the internal structure of the construct. The first model **(A)** corresponds to a multidimensional structure of six oblique factors, because it evidences strong covariances; likewise, this model presents excellent factor loadings between 0.75 and 0.94. The global evaluation of the model according to the fit indices is very satisfactory (χ2 = 730.643, gl= 237, p = 0.000, CFI = 0.985, TLI = 0.983, RMSEA = 0.062 [0.057, 0.067], SRMR = 0.030). The second model **(B)** offers a second-order internal structure, where the second-order construct Teaching explains with high weights (>0.92) the first-order factors: competence exploration, explanation of criteria and illustration; similarly, the second order construct Formative assessment explains with high weights the behavior of the first-order factors: supervision of practice, feedback and evaluation. The global fit indices of the second-order model are also very good (χ2 = 763.713, gl= 245, *p* = 0.000, CFI = 0.984, TLI = 0.982, RMSEA = 0.063 [0.058, 0.068], SRMR = 0.032).

These results support not only the possibility of measuring the six criteria of teacher didactic performance, but also the second-order factors and the construct in general, as evidenced by the high covariations of the two factor models.

Table 2 shows the reliability results estimated with McDonald's omega (ω) for categorical items. The omega coefficients were greater than 0.90 for the factors (didactic performance criteria), as well as for the second-order constructs and for the general scale. The reliability estimated with the H coefficient also denotes the presence of high precision for the measurement of the scale constructs.

**Table 2:** Reliability of the student rating scale on the teacher's didactic performance.

**Criteria**	**ω**	***H***
Competence exploration	0.908	0.917
Explanation of criteria	0.942	0.949
Illustration	0.929	0.935
Supervision of practice	0.942	0.943
Feedback	0.957	0.963
Evaluation	0.951	0.955
Teaching	0.974	0.978
Formative assessment	0.983	0.984
General scale	0.989	0.991

### Evidence of validity and reliability of the students' self-assessment scale

Figure 4 presents the results of the evaluation of two internal structure models for student didactic performance. The six-factor multidimensional model **(C)** presents excellent factor loadings (> 0.80) and strong interfactor covariances (>0.70), and the global fit indices support the validity based on the internal structure of the construct (χ2 = 885.893, gl= 237, *p* = 0.000, CFI = 0.977, TLI = 0.973, RMSEA = 0.071 [0.066, 0.076], SRMR = 0.038). The second model supports the validity of a second-order factor structure (χ^2^ = 988.711, gl= 245, p = 0.000, CFI = 0.973, TLI = 0.970, RMSEA = 0.075 [0.070, 0.080], SRMR = 0. 042), where the second-order construct named Learning explains with high weights the existence of three primary factors such as Pre-current for learning, identification of criterion and illustration-participation; likewise, the following second-order factor named Improvement-Application, is also configured with the presence of three primary factors such as pertinent practice, feedback-improvement and evaluation-application.

According to Table 3, McDonald's omega internal consistency coefficients are greater than 0.90 for the primary, second order factors and general scale. Likewise, the H coefficient for the reliability of the constructs is high (>0.90). This implies that the student didactic performance self-assessment scale allows high precision measures for the instrument scores.

**Table 3:** Reliability of the student self-assessment scale on their didactic performance.

**Criteria**	**ω**	***H***
Precurrent for learning	0.939	0.949
Identification of criteria	0.918	0.926
Illustration-participation	0.938	0.939
Pertinent practice	0.937	0.939
Feedback-improvement	0.942	0.942
Evaluation-application	0.931	0.934
Learning	0.976	0.979
Improvement-Application	0.978	0.979
General scale	0.988	0.989

### Relationship between teacher and student didactic performance in each pair of criteria

**Figure 5:**
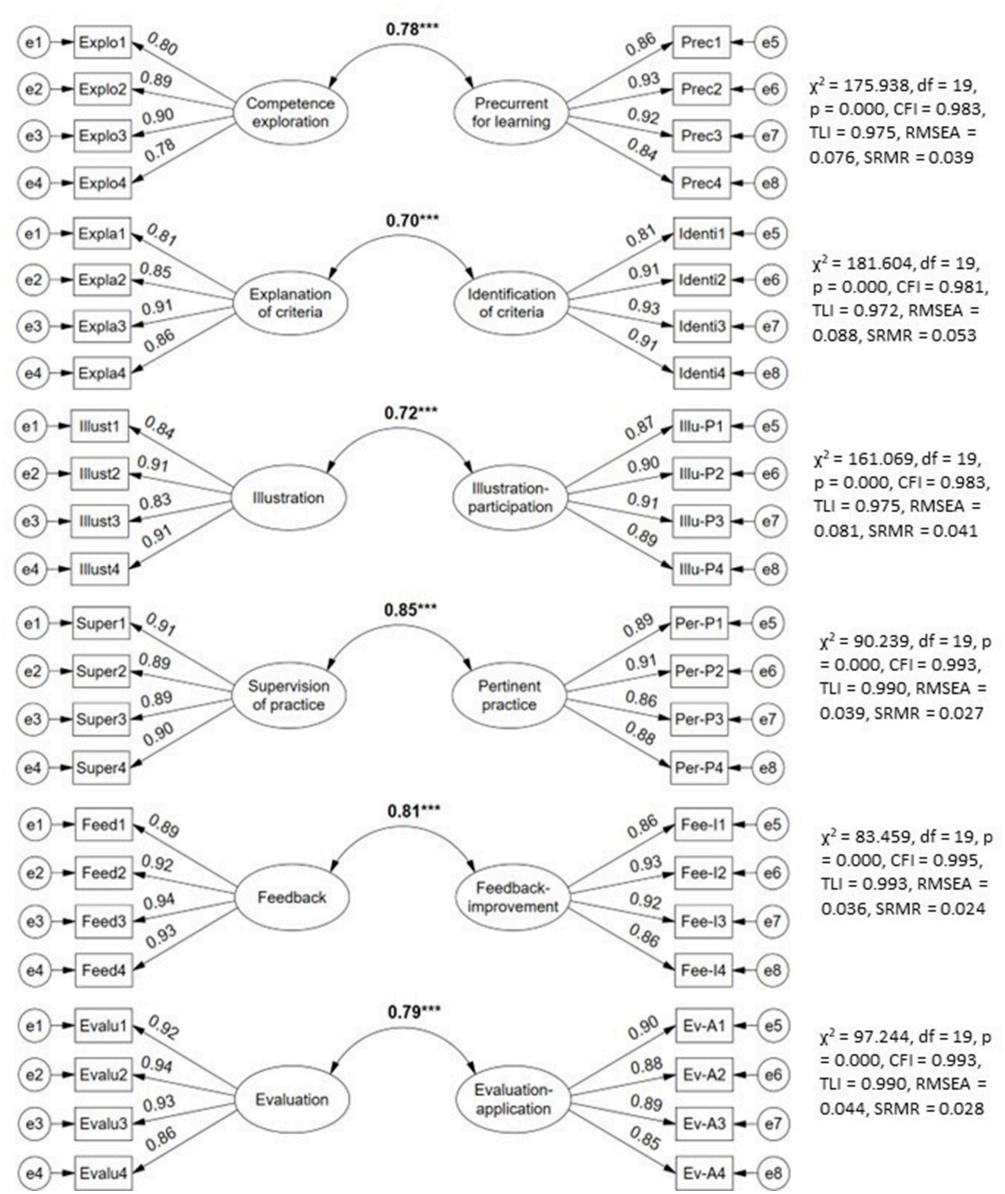
Relationship between didactic performance criteria teacher-students.

All latent co-variances are strong, positive and with large effect sizes (≥0.70) between each pair of teacher-student didactic performance criteria (see Figure 5). Each latent relationship between the criteria evaluated with structural equation modeling presented satisfactory fit indices given that CFI and TLI exceeded the recommended cutoff value (>0.95), SRMR almost entirely less than 0.04 (good fit) and RMSEA denoting between adequate ( ≤ 0.08) and good fit (< 0.05).

### Predictive models of student teaching performance variables

To examine whether teaching and formative assessment (second-order factors of teacher didactic performance) are factors that can predict changes in learning and improvement-application, two linear regression models were tested. As shown in Table 4, model 1 is statistically significant (*p* < 0.001) in predicting student learning as a function of teaching and formative assessment with an explained variance of 54%; likewise, model 1 highlights that the teaching factor presents greater predictive capacity according to the standardized beta coefficient (β = 0.42). On the other hand, model 2 shows that the capacity for improvement-application of what has been learned is predicted in 61% by the factors teaching and formative assessment. In model 2, the β coefficient shows that formative assessment has greater predictive power for improvement-application in contrast to the teaching factor.

**Table 4:** Prediction of second-order factors of student didactic performance.

**Model 1**		**Dependent variable: learning**						
		***B***	***SE***	β	***t***	***p***	**95% CI**	
(Constant)	2.223	0.193		11.543	0.000	1.845	2.602
Teaching	0.369	0.061	0.417	6.080	0.000	0.250	0.489
Formative Evaluation	0.293	0.060	0.336	4.890	0.000	0.176	0.411
F _(2, 538)_ = 315.911, p = 0.000, R = 0.735, R^2^= 0.540								
**Model 2**		**Dependent variable: improvement-application**						
	***B***	***SE***	β	***t***	***p***	**95% CI**	
(Constant)	2.078	0.180		11.557	0.000	1.725	2.431
Teaching	0.192	0.057	0.213	3.390	0.001	0.081	0.304
Formative Evaluation	0.520	0.056	0.584	9.279	0.000	0.410	0.630
F _(2, 538)_ = 425.577, p = 0.000, R = 0.783, R^2^= 0.613								

**Figure 6:**
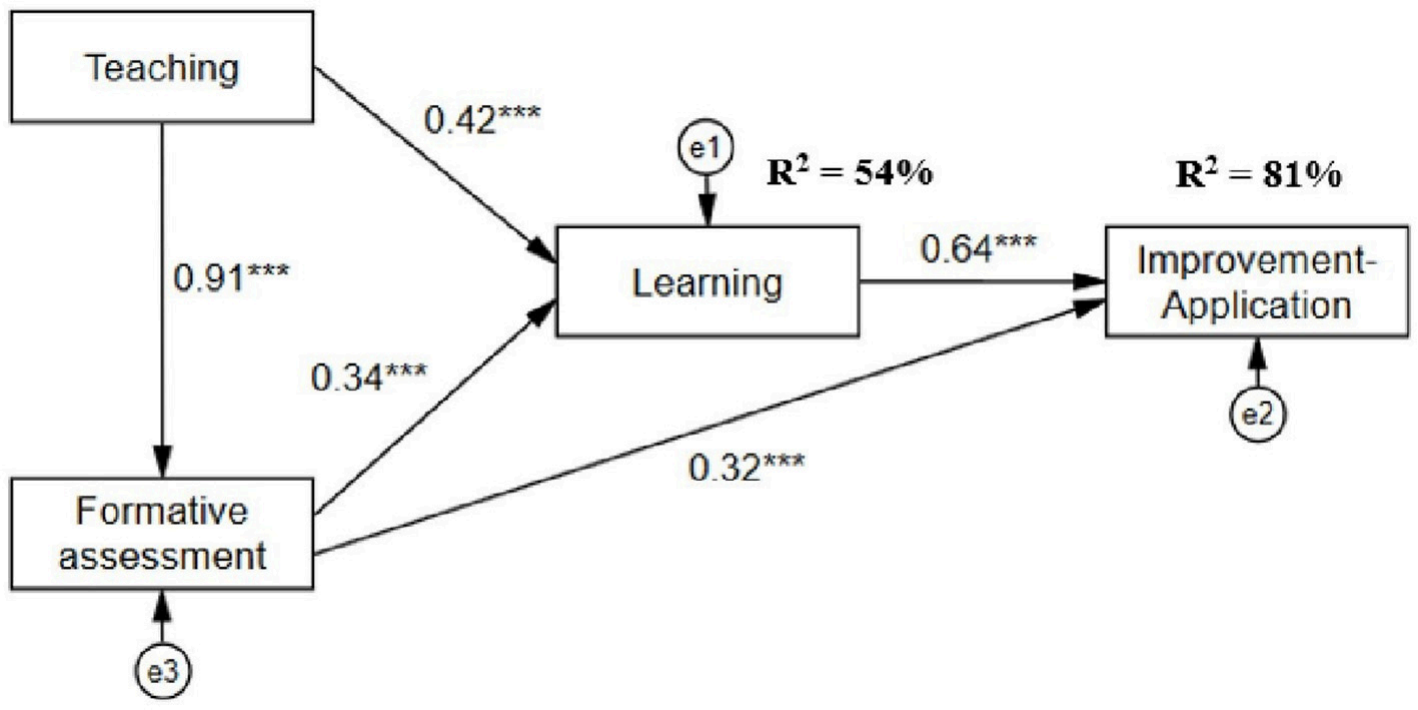
Path analysis of of the predictive model of student didactic performance.

As shown in Figure 6, teaching and formative assessment have a positive and direct influence on student learning (*R*^2^ = 54%). However, teaching and formative assessment have significant and indirect effects on improvement-application (*R*^2^ = 81%), with learning acting as a mediator. This Path Analysis model is valid according to the fit indices: χ2 = 1.681, gl = 1, *p* = 0.195, CFI = 1.00, TLI = 0.998, RMSEA = 0.036, SRMR = 0.003, GFI = 0.998, AGFI = 0.984.

## Discussion and conclusion

The first finding shows that students self-assessed behavior regarding their performance in each of the six teaching criteria corresponds functionally with the perceived didactic performance of their teachers. This is empirical evidence of what Irigoyen et al. (2011b) theoretically stated that, “the student identifies the level of correspondence shared by two or more situations that are morphologically different but functionally regulated in an equivalent way” (p. 237).

The results of the present work also coincide with the findings of significant relationships between teacher performance and student performance, as perceived by the student body, in each pair of domains or criteria of didactic interactions in the context of teaching in biological sciences (Velarde-Corrales and Bazán-Ramírez, 2019; Bazán-Ramírez et al., 2023). In psychology classes, the functional relationship, at least in the perception of the student body through self-report scales, between the didactic performance of the teacher and the didactic performance of the student body is proven. In other words, the didactic performance of the students corresponds to the performance of the teacher in a specific didactic area or criterion, in a learning situation. This learning situation specifies “the characteristics of the actions or behaviors that functionally correspond in that situation” (Ribes, 2008).

On the other hand, the validity based on the internal structure of the construct has been corroborated for self-reports of teaching performance, and the convergent and divergent validity of the six teaching performance criteria for both teachers and students in psychology classes has been confirmed, coinciding with the results obtained with biology students (Bazán-Ramírez et al., 2023) and Education Sciences students (Bazán-Ramírez et al., 2022b). One aspect to highlight in the present study, in addition to confirming the significant functional relationships between each pair of teaching criteria reported with undergraduate Biology students. Furthermore, better association indexes were obtained between each pair of didactic performance criteria.

Another relevant aspect was to confirm the second-order factors in the didactic interactions of teaching and learning in the discipline of psychology. The second-order factors “teaching and formative assessment” correspond to teacher performance, and for student performance, the second-order factors are “learning and improvement-application.” This expands on the first model of teaching and formative assessment reported by Bazán-Ramírez et al. (2022a), which only focused on the didactic behaviors of teachers. These second-order latent factors facilitate the identification of instructional variables in didactic interactions that influence learning processes and academic achievement (Carroll, 1963; Elliott, 2015) which constitute learning opportunities within a teaching model based on interbehavioral psychology (Carpio et al., 1998; Irigoyen et al., 2011a; Kantor, 1959; Kantor and Smith, 1975; Silva et al., 2014; Ribes, 1993; Ribes et al., 1996).

The second objective was to determine the effect of second-order variables of teacher performance as perceived by students (teaching and formative assessment) on second-order variables of student performance (learning and improvement-application). The results of the linear regression and path analysis models indicated that teaching and formative assessment have a direct impact on student learning and improvement-application.

Likewise, the assumed model of the influence of teaching and formative assessment by teachers on student learning and improvement-application was verified. In this sense, teaching performance in terms of improvement and application was indirectly affected, in that the “learning” factor is an important mediator that explains the effect of teaching and teacher assessment on student improvement and application of what has been learned.

Based on this data, it can be inferred that the teacher's educational strategy has a direct impact on student learning; this indicates that student performance in terms of learning depends on the teacher's didactic performance, i.e., it is functionally adjusted to the teacher's didactic criteria and practices. Furthermore, the formative assessment deployed by the teacher also has a greater influence on student learning and on the improvement and application of the student's part, which highlights the importance of formative assessment, both in the learning processes themselves and in feedback and supervision practices to encourage students to improve their skills and abilities, so that they can apply them in contexts other than learning situations (Capa-Luque et al., 2025).

The results of the present study extend the findings reported with graduate students in Educational Sciences by Bazán-Ramírez et al. (2022a), but better specify in the teaching-learning of the discipline of Psychology, the effect of two instructional variables that correspond to the didactic performance of the teacher (Teaching and Formative assessment), on two didactic performance variables of the student body: learning and improvement-application. However, as mentioned above, the results of the present study showed evidence of the effect of the teaching and formative assessment variables on the didactic performance of students and their impact on the educational process.

The reported data coincide with the finding of Montes de Oca et al. (2023) that the learning strategies used by Psychology students are linked to the didactic performances of the subject's faculty. The teacher's ability to implement varied strategies in the classroom, as detailed in the categorization of Ochoa et al. (2022), reflects different dimensions of teaching performance that students value, including adequate planning, knowledge management, and the use of technologies. They also coincide with (Arce-Saavedra and Blumen 2022) statement that teacher self-efficacy and innovative performance influence the quality of teaching. In a similar line, Martínez et al. (2022) indicated that the application of didactic strategies based on formative assessment generates a significant impact on learning, especially in virtual environments; that is, formative assessment processes increase student motivation and autonomy, promoting active participation in their learning. Research by Martín-Párraga et al. (2023) confirms that teachers who incorporate digital resources in their classes tend to be perceived as more competent, which has a direct impact on student satisfaction.

Along the same lines as the results presented above, the work of Cuellar-Quispe et al. (2023) highlights that students' perception of teachers' performance is related to specific evaluation criteria. This is supported by the notion that good communication and democratization of the teaching-learning process strengthen the relationship between teacher and student.

In the context of the present study, the didactic performance of the teacher seems to influence the way in which students engage with the material. The practice of innovative methods and learning-centered and participatory approaches are associated with student learning, increasing student interest and, therefore, with improvements in their academic performance. On the impact of feedback on the quality of learning, a relevant aspect in the analysis of the results is the role of formative assessment, which includes feedback. The findings of this study indicate that constant and relevant feedback from teachers not only improves performance, but also has a favorable impact on student self-efficacy. This aligns with the results of Bazán-Ramírez et al. (2025) who suggest that students who receive direct and effective teaching are more likely to take responsibility for their own learning. Likewise, interviews conducted by Martínez et al. (2022) on formative assessment revealed that teachers who engage in the feedback process generate an environment conducive for students to feel comfortable sharing their concerns and questions. This link can foster the creation of a space where mistakes are seen as learning opportunities, which in turn promotes a more positive approach to the educational process.

There are two strengths of this study: the first strength has to do with the validity of measurement constructs with first and second order latent factors to characterize teacher-student performance criteria in didactic interactions in teaching-learning of psychology, under a substantive theory of psychological and pedagogical construct measurements, and not only based on the psychometric properties of the scales. The second strength is to have obtained co-variation models between first-order factors that denote, at the self-report level, functionally corresponding pairwise relationships between teacher and student performance. Similarly, we have obtained a model with good goodness of fit to explain the didactic performance of students in the improvement of their learning based on teacher feedback, and its application in various situations of evaluation and necessary performance.

However, we can identify several limitations in our study. The first has to do with the use of self-reports to inquire about past didactic interactions. In addition to having a solid substantive theory from the psychological, self-report questionnaires can be suitable complements to observational studies of didactic performances, such as those reported by Neves-Balan et al. (2022) and by (Velarde-Corrales and Bazán-Ramírez 2019). A second limitation is properly, the use of students' self-reports to assess the didactic performance of their teachers. These could reveal subjectivation motivated by other patterns of the teacher or of the students themselves, and even of reciprocity (Alquraan et al., 2024; León, 1978). It would be convenient in studies of this type to consider the proposal of Cook et al. (2024) to control biases in teacher evaluation, especially when the evaluators are their students.

A third important limitation is related to the use of a non-experimental design. In this sense, the causal relationships correspond to statistical criteria specific to the Path Analysis technique of the SEM methodology, which allows modeling causal relationships by taking into consideration three conditions (Byrne, 2010; Keith, 2019): (1) the relationships between the variables are functional, (2) there is a logical temporality between the variables (the cause precedes the effect in time), and (3) the relationship between the variables is not spurious. To strengthen the evidence found with Path Analysis, it is suggested to replicate studies with other samples, especially with random samples. Finally, another limitation that affects the generalizability of results corresponds to the use of non-probability sampling.

## Conclusions

The first conclusion is that the results of this research confirm the correspondence between the teacher's assessment and the student's assessment in each of didactic performance criteria. The results and statistical analyses confirm the possibility of measuring the six criteria of didactic performance, as well as the second-order factors and the construct in general.

Second-order factors in didactic interactions are confirmed. Teaching and Formative Assessment for teacher performance; Learning and Improvement-Application for student performance. Thus, expanding the first model of Teaching and Formative Assessment.

The second conclusion is that teaching and formative assessment (second-order factors of the teacher's didactic performance) are factors that predict changes in student learning and improvement-application, fulfilling a mediating role, learning.

## Data Availability

The raw data supporting the conclusions of this article will be made available by the authors, without undue reservation.
