# Supplementary material for: Criteria of teacher and students's didactic performances in psychology: the Peruvian university students' perceptions
Source: Front Psychol. 2026 Feb 16;17:1751054. doi: 10.3389/fpsyg.2026.1751054 (PMC12950763; doi:10.3389/fpsyg.2026.1751054)
Supplement: Supplementary file 1 [file Supplementary_file_1.docx]

**Appendix 1**

STUDENT RATING SCALE ON THEIR TEACHER'S DIDACTIC PERFORMANCE IN CLASSES

|  | **Never**  **0** | **Almost Never**  **1** | **Almost Always 2** | **Always**  **3** |
| --- | --- | --- | --- | --- |
| **Competence Exploration** |  |  |  |  |
| At the beginning of this course, the teacher assessed my prior knowledge, either orally and/or through a written questionnaire. |  |  |  |  |
| At the beginning of each class, the teacher explored my skills and knowledge of the topic that was going to be covered in the class. |  |  |  |  |
| At the beginning of each learning unit, the teacher assessed my level of mastery of the skills outlined in the Syllabus. |  |  |  |  |
| The teacher adapted the course content according to the level of our prior knowledge and skills. |  |  |  |  |
| **Criteria Explanation** |  |  |  |  |
| When starting a learning unit, the teacher explained what the expected achievement is in that unit (The learning objective to be achieved in the course). |  |  |  |  |
| The teacher explained the criteria we had to meet to carry out an activity or task. |  |  |  |  |
| The teacher explained the criteria and requirements I needed to meet to complete an exercise in class, or a course practice. |  |  |  |  |
| The teacher clearly explained the achievement criteria that the student had to meet in each class. |  |  |  |  |
| **Illustration** |  |  |  |  |
| The instructor provided examples of how to develop a task or practice. |  |  |  |  |
| The instructor solved problems in front of the students based on the topic presented and asked us to do the same. |  |  |  |  |
| The instructor described how a professional in the field I study would solve a relevant problem in the course. |  |  |  |  |
| Regarding a solution to a problem, the instructor described the solution process and the reasons for applying that solution. |  |  |  |  |
| **Practice Supervision** |  |  |  |  |
| During the course activities, the teacher supervised my performance in class exercises. |  |  |  |  |
| The teacher helped us solve problems and exercises in class. |  |  |  |  |
| The teacher controlled the rules and conditions of the activities, to ensure that the students learned effectively. |  |  |  |  |
| All students receive teacher supervision in course activities. |  |  |  |  |
| **Feedback** |  |  |  |  |
| The professor provided feedback on the exercises in class, pointing out our successes and failures. |  |  |  |  |
| The teacher pointed out my mistakes and showed me the way(s) in which I can improve my performance. |  |  |  |  |
| The teacher showed me different ways I can meet the criteria for his class activities. |  |  |  |  |
| The professor gave us suggestions and guidance to correct and improve our skills in the course. |  |  |  |  |
| **Assessment** |  |  |  |  |
| The professor periodically assessed us on my theoretical knowledge or basic foundations of the subject. |  |  |  |  |
| The professor conducted applied assessments and the solution of practical problems derived from the subject. |  |  |  |  |
| The teacher evaluated us according to the learning objectives he outlined at the beginning of the course, which are included in the course outline. |  |  |  |  |
| The professor assessed my ability to integrate knowledge from other courses with the current course. |  |  |  |  |

STUDENT SELF-ASSESSMENT SCALE ON THEIR DIDACTIC PERFORMANCE

|  | **Never**  **0** | **Almost Never**  **1** | **Almost Always 2** | **Always**  **3** |
| --- | --- | --- | --- | --- |
| **Precurrents for learning** |  |  |  |  |
| At the beginning of this course I demonstrated my previous knowledge, according to the evaluation given by the teacher. |  |  |  |  |
| At the beginning of each class, I responded and showed the teacher my skills and knowledge of the topic to be developed in the class. |  |  |  |  |
| I demonstrated my previous competencies and skills, at the beginning of each instructional unit, according to the syllabus. |  |  |  |  |
| I answered the teacher's questions and/or formulated questions about concepts related to the topic I was going to develop. |  |  |  |  |
| **Identification of criteria** |  |  |  |  |
| I identified what the achievement (competencies or learning objectives) was that the teacher expected me to achieve in that learning unit. |  |  |  |  |
| I performed the activity or task, meeting the criteria explained by the teacher. |  |  |  |  |
| When I did some exercise in class or developed some practice in the course, I met the criteria previously established by the professor. |  |  |  |  |
| I applied the achievement criteria established by the teacher, for my learning in the class. |  |  |  |  |
| **Illustration - Participation** |  |  |  |  |
| I developed my tasks or practices, following the examples given by the teacher on how to perform the task or practice. |  |  |  |  |
| I performed in-class activities or homework, following the model that the teacher developed. |  |  |  |  |
| I solved a problem as a professional in my career would do it, or as close as possible to how such a professional would do it. |  |  |  |  |
| About a solution to a problem, I describe the solution process and why apply that solution. |  |  |  |  |
| **Relevant practice** |  |  |  |  |
| During the classes or internships, I developed my activities under the guidance and supervision of the teacher. |  |  |  |  |
| I solved problems and exercises in class, under the guidance of the teacher. |  |  |  |  |
| I learned effectively by adjusting to the rules and conditions that the teacher established in class. |  |  |  |  |
| All students have been supervised by the teacher during the course activities. |  |  |  |  |
| **Feedback - Improvement** |  |  |  |  |
| I corrected my performance in order to develop the class activities in a pertinent manner, based on my successes and failures pointed out by the teacher. |  |  |  |  |
| With the support of the teacher I identified and learned how I should solve problems and perform tasks. |  |  |  |  |
| I performed the class activities including the different forms taught by the teacher. |  |  |  |  |
| I corrected my work as directed by the teacher to improve my performance and meet the criteria outlined for the assignment. |  |  |  |  |
| **Evaluation - Application** |  |  |  |  |
| I demonstrated that I have mastered the theoretical knowledge or basic fundamentals of the subject. |  |  |  |  |
| Developed the evaluations by applying my knowledge in the solution of practical problems. |  |  |  |  |
| I have demonstrated knowledge and competencies according to the learning objectives stated in the course syllabus. |  |  |  |  |
| Integrated knowledge from other subjects to answer questions on the topic being developed. |  |  |  |  |
